# Mercurial Collodion in the Removal of Syphilic Patches of Discoloration

**Published:** 1867-01

**Authors:** 


*Mercurial Collodion in the Removal of Syphilic Patches of Discoloration.*—M. Leclerc states in the *Presse Medicale*

*Belge*, that a patient of his having tried alkaline, vapor, and sea-baths for the removal of those patches which appear on the skin of syphilitic patients, without effect, he recommended her to apply the following lotion, which removed them in a few days: Corrosive sublimate, fifty centigrammes; collodion fifteen grammes.—*Richmond Med. Jour.*

